# Change of Fibroblast Growth Factor 21 Level Correlates with the Severity of Diabetic Sensory Polyneuropathy after Six-Week Physical Activity

**DOI:** 10.31083/j.rcm2305160

**Published:** 2022-04-28

**Authors:** Ágnes Molnár, Anita Szentpéteri, Hajnalka Lőrincz, Ildikó Seres, Mariann Harangi, Zoltán Balogh, Péter Kempler, György Paragh, Ferenc Sztanek

**Affiliations:** ^1^Division of Metabolic Disorders, Department of Internal Medicine, University of Debrecen Faculty of Medicine, 4032 Debrecen, Hungary; ^2^Doctoral School of Health Sciences University of Debrecen, 4032 Debrecen, Hungary; ^3^First Department of Internal Medicine, Semmelweis University Faculty of Medicine, 1085 Budapest, Hungary

**Keywords:** diabetic neuropathy, current perception threshold, fibroblast growth factor-21, inflammation, oxidative stress, physical activity

## Abstract

**Background:**

Diabetic neuropathy (DN) is a very frequent microvascular complication of type 2 diabetes mellitus (T2DM). Obesity and physical inactivity are well-known risk factors for T2DM. Fibroblast growth factor 21 (FGF21) is a liver-secreted hormone with several beneficial effects on obesity-related metabolic disorders. We aimed to investigate the effect of short-term physical activity on the levels of FGF21, and its correlation with the severity of peripheral sensory polyneuropathy in T2DM patients.

**Methods:**

Thirty patients with DN were enrolled in the study, compared to age- and gender-matched controls. We conducted a six-week aerobic training program, which meant treadmill and cycle ergometers three times a week. Anthropometric and laboratory parameters were measured for each patient before and after intervention. Serum levels of FGF21, TNF-alpha, irisin, leptin and adiponectin were measured by ELISA. The sensory perception threshold (CPT) was quantitatively measured using Neurometer®.

**Results:**

We found significant decreases in BMI, waist circumference, HbA1c and TNF-alpha levels. From baseline to six-week follow-up, FGF21 levels were significantly increased in DN patients. Significant negative correlations were shown between the changes in FGF21 levels and BMI, between changes in FGF21 and the improvement of CPT values, and between the changes in FGF21 and TNF-alpha levels. There was no difference in irisin, adiponectin and leptin levels in DN patients after aerobic training program.

**Conclusions:**

The physical activity may increase the level of FGF21 in T2DM patients with neuropathy. Our results highlight the importance of regular physical activity in the treatment of diabetic neuropathy.

## 1. Introduction

Diabetic neuropathy (DN) is a quite frequent microvascular complication of type 2 diabetes mellitus (T2DM), which is often diagnosed as distal sensory polyneuropathy. The development of diabetic neuropathy is a multifactorial progression and the precise pathomechanism is not fully clarified. Impaired glucose metabolism is associated with increased reactive oxidative species production, mitochondrial dysfunction and accumulation of glycolytic intermediates that stimulate other metabolic or non-metabolic pathways instead of switching to glycolysis, resulting in activation of polyol, hexosamine, and protein kinase-C pathways. Chronic hyperglycaemia induces the production of reactive oxygen species and increases oxidative stress, which plays an important role in the development of mitochondrial dysfunction in distal sensory polyneuropathy [1].

Previous research indicates that physical activity may improve the neurological function and impaired nerve conduction in DN [2]. Physical activity and regular exercise are effective interventions to reduce the development and progression of diabetic neuropathy. Moreover, it is important that patients with polyneuropathy perform regular physical activity under appropriate health and physiotherapy supervision. Uncontrolled exercise due to peripheral nerve damage may contribute to the development of ulcers and diabetic foot syndrome, as the insensitive foot is unable to mediate the nociceptive stimuli required to elicit protective behavior, which affects peripheral sensation and vasomotor regulation of foot circulation [3]. However, a recent meta-analysis has demonstrated that controlled exercise is a beneficial non-pharmacological intervention for the management of diabetic foot, especially in increasing nerve velocity conduction in the lower extremities. Exercise in diabetic patients may have additional benefits, such as skin sensitivity and intraepidermal nerve density, which may delay the normal course of diabetic peripheral neuropathy, as well as delay skin damage and ulceration [4]. According to the statement of American Diabetes Association, structured lifestyle modification which include at least two and a half hour per week physical activity and dietary changes, are also recommended to delay the progression of microvascular complications in T2DM. Moreover, all diabetic patients with or without peripheral neuropathy should perform both aerobic and resistance training for optimal glycemic control [5].

Regular exercise in diabetes has a favorable effect on insulin resistance, especially in the liver and muscle tissue. Several factors may contribute to insulin resistance in liver, including the formation of reactive oxygen species, genetic factors, aging, and mitochondrial dysfunction. Fibroblast growth factor 21 (FGF21) is a liver-secreted hormone with several beneficial effects on obesity-related metabolic disorders and insulin resistance. FGF21 enhances glucose uptake and oxidation in an insulin-independent manner by inducing the expression of glucose transporter-1 in adipocytes and skeletal myocytes [6]. Previous experimental and human studies have shown that physical activity may increase the serum levels of FGF21 in T2DM [7]. According to a recent meta-analysis, acute exercise significantly increased the serum concentration of FGF21, regardless of body weight and obesity level [8]. However, it is still not clarified how FGF21 may improve the process of mitochondrial oxidation [9].

It is well known that skeletal muscle produces and releases cytokines during exercise, which are termed myokines. Irisin is a myokine expressed by physical activity with insulin-sensitizing properties and derived from the C-terminal cleavage of the fibronectin type III domain containing 5 (FNDC5) transmembrane proteins [10]. This proteolytic process is mediated by the peroxisome proliferator-activated receptor-gamma coactivator-1-alpha (PGC-1α). Irisin/FNDC5 acts on skeletal muscle during exercise, resulting in glucose and fatty acid uptake, as well as increased energy expenditure and induces endogenous oxidative stress through the initiation of several metabolic genes involved in the regulation of the mitochondrial bioenergetic process [11].

In addition to energy storage, adipose tissue produces a variety of adipocytokines, including leptin, adiponectin and others, thus having potential endocrine function. Leptin may directly improve insulin resistance in diabetic mice by increasing the oxidation of free fatty acid (FFA) [12]. Previous research has shown that decreased circulating adiponectin is positively associated with insulin resistance and the markers of cardiovascular disease in T2DM subjects [13]. Furthermore, an increased proinflammatory response is observed in leptin resistance during obesity and physical activity may reduce inflammation by improving leptin resistance in T2DM [14]. Tumor necrosis factor alpha (TNF-alpha) is a pro-inflammatory adipokine associated with insulin resistance and β cell failure in T2DM and obesity [15]. High level expression of TNF-alpha induces phosphorylation of the insulin receptor substrate 1 and thus prevents the interaction of insulin with an insulin receptor [16]. The anti-inflammatory properties of adiponectin may play a central role in slowing the progression of atherosclerosis in T2DM and may have a beneficial effect on insulin resistance by inhibiting TNF-alpha-induced activation of NF-κB in endothelial cells [17]. Moreover, TNF-alpha improves activity of hormone sensitive lipase via its autocrine and paracrine effects, therefore the release of FFA is increasing into circulation [15]. According to previous studies, diabetic neuropathy is associated with elevated leptin concentrations and activation of the TNF-alpha system [18, 19]. However, the effects of increased FGF21 and irisin production on various adipokines and inflammatory markers as a result of physical activity have not been studied in diabetic neuropathy.

In the current study, we aimed to investigate the changes of FGF21 level and their relationships with other inflammatory markers and adipokines in T2DM patients with distal sensory polyneuropathy after six-week of aerobic exercise training program. We hypothesized significant correlations between the changes of FGF21 level and the severity of peripheral sensory neuropathy in T2DM patient after physical activity.

## 2. Materials and Methods

### 2.1 Study Population

Our study group included 30 adult individuals with T2DM and distal sensory polyneuropathy (9 men and 21 women; the mean age: 61.97 ± 8.1 years; mean duration of T2DM was 10.3 ± 3.7 years, and mean duration of DN: 8.7 ± 5.6 years. Besides, 32 age- and gender-matched T2DM patientswithout neuropathy were also enrolled as controls (10 men and 22 women; the mean age was 64.37 ± 6.52 years; mean duration of T2DM was 10.9 ± 4.1 years). All patients underwent to oral antidiabetic therapy containing metformin, sulfonylurea and/or DDP4-inhibitors, whereas participants who use insulin were excluded from this study. Presence of diabetic proliferative retinopathy, diabetic nephropathy (eGFR <60 mL/min/1.73 $m^{2}$ and/or persistent albuminuria) as well as type 1 diabetes were further exclusion criteria. Patients in exercise group had normal resting electrocardiogram tests, no ischemic heart disease, no symptoms of peripheral arterial disease and limit values of ankle/brachial index (abnormal values for the resting ankle-brachial index are 0.9 or lower and 1.40 or higher). Subjects with prior cardiovascular disease, established coronary artery disease or myocardial infarction, severe congestive heart failure (NYHA class III–IV), pregnant women, smokers, subjects with established malignancy were not included in the study. Furthermore, Subjects with alcohol and drug dependence, known liver diseases, autoimmune and endocrine disease, neurological and haematological disorders were also excluded. Participants were recruited from the Diabetic Neuropathy Center, Department of Internal Medicine, University of Debrecen, Debrecen, Hungary. Written informed consent was provided from all patients. The protocol was approved by the institutional ethical committee and the Medical Research Counsil (ETT-TUKEB code: 5287-2/2019/EKU, date of approval: 07/03/2019). The study was conducted in accordance with the ethical principles for medical research (Declaration of Helsinki).

### 2.2 Study Design

**Fig. 1.**
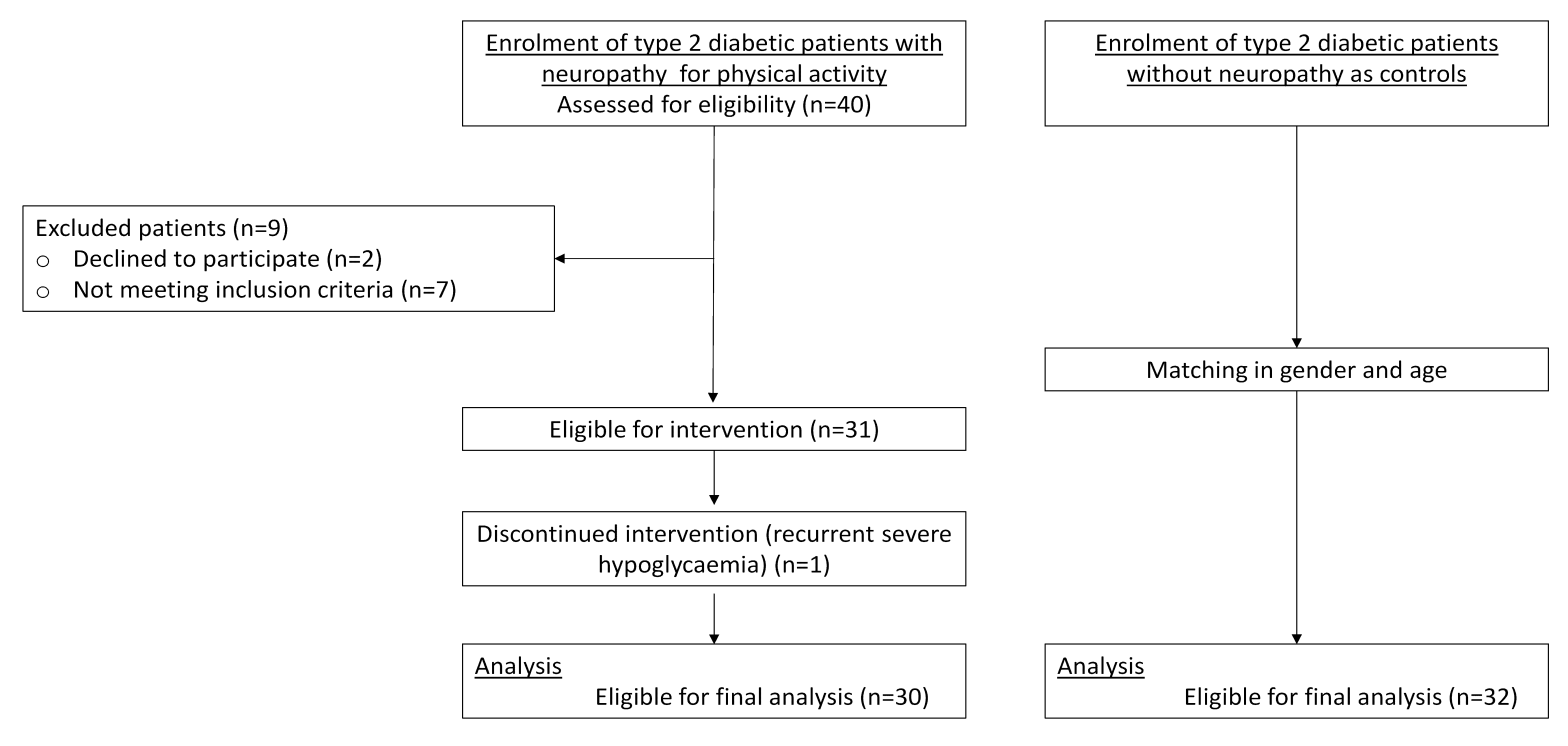
**Study design flowchart of type 2 diabetic patients with and without neuropathy**.

Study design flowchart of diabetic patients with neuropathy are depicted on Fig. 1. After final enrolment, DN patients were instructed to march with trekking poles and the aerobic exercise training program was supervised by a physiotherapist and corrected if needed. Glucose levels were determined immediately after the training and one hour later. If significant drop of serum glucose levels were measured, antidiabetic therapy has been modified according to the needs of exercise. The subjects had to perform the exercises for 6 weeks, 3 days a week, occasionally for 70 minutes. The exercise program whose duration has progressed gradually (from 50% to 80% of maximum heart rate), included 10 minutes of stretching movements until warm-up, then 50 minutes of aerobic training (treadmill and bicycle ergometers), followed by 10 minutes of relaxation activity to cool down. Before and after the intervention, we measured the cardio fitness levels by ${\mathrm{VO}}_{\text{2max}}$ (mL/kg/min) using the Rockport 1600 m walking test. Estimation of ${\mathrm{VO}}_{\text{2max}}$ over a timed one-mile walk, including age, gender, body weight and heart rate at the end of the walk test [20]. The body mass index (BMI) and heart rate (monitored with PolarA300, 17954515.02 ENG 04/2016, China) of the patients were also measured before and after the exercise training program. After 6 weeks of supervised training, all patients underwent blood tests during outpatient care. All patients with neuropathy underwent blood test and neurophysiological examination for objective evaluation of sensory neuropathies using the Neurometer® (Neurotron Inc., Baltimore, MD, USA, 2002). current perception threshold testing during the outpatient visit. The control subjects were not exposed to the exercise training program, only were used as a benchmark for the comparison of results.

### 2.3 Blood Sampling

Peripherial blood samples were withdrawn after 12-hour overnight fast into Vacutainer® tubes. After centrifugation, routine clinical parameters (i.e., creatinine, uric acid, glucose, hemoglobin A1c—HbA1c, triglyceride, total cholesterol, low-density lipoprotein-cholesterol—LDL-C, high-density lipoprotein-cholesterol—HDL-C) were measured with Cobas 6000 autoanalyzer (Roche Ltd., Mannheim, Germany) in the Department of Laboratory Medicine, University of Debrecen, Faculty of Medicine, Debrecen, Hungary. High-sensitivity C-reactive protein (hsCRP), ApoA1, ApoB and Lp (a) levels were determined by immune-turbidimetric assays. Total cholesterol (TC), triglyceride, HDL-C and LDL-C levels were measured by enzymatic colorimetric tests. All measurements were performed according to the manufacturers’ recommendation. Samples for further ELISA determinations were kept at –80 °C.

### 2.4 FGF21 Measurement

Serum FGF21 concentration was detected by commercially available sandwich enzyme immunoassay (Human FGF21 ELISA, Biovendor, Brno, Czech Republic) with intra-assay coefficient variations ranging from 1.6 to 2.4% and inter-assay coefficient variations ranging from 3.1 to 3.5%, respectively. Determination of FGF21 level in sera were performed according to the manufacturer’s recommendation. The values were presented as pg/mL with the assay range of 30–1920 pg/mL and 7 pg/mL limit of detection.

### 2.5 TNF-alpha Measurement

We measured serum levels of TNF-alpha by using commercially available sandwich enzyme immunoassay according to the recommendations of the manufacturer (R&D Systems Europe Ltd., Abington, England). The intra-assay coefficient variations were 1.9–2.2% and inter-assay coefficient variations were 6.2–6.7%, respectively. The values were presented as pg/mL with 0.156–10 pg/mL assay range and 0.049 pg/mL sensitivity.

### 2.6 Measurement of Irisin Levels

Circulating irisin were measured by competitive ELISA (Human Irisin ELISA, Biovendor, Brno, Czech Republic). The calibration range was 0.001–5 μg/mL, as well as the coefficient variations of the intra- and inter-assay for measurement of irisin were 4.8–7.9% and 8.0–9.7%, respectively and the lowest level of irisin can be measured by this assay is 1 ng/mL according to the instructions of the manufacturer.

### 2.7 Determination of Adiponectin and Leptin Levels

Serum adiponectin and leptin levels were measured with ELISA (Human Total Adiponectin/Acrp30 Quantikine and Human Leptin Quantikine Immunoassays, R&D Systems Europe Ltd., Abington, England). The coefficient variations of the intra- and inter-assay for measurement of total adiponectin were 2.5–4.7% and 5.8–6.9%, respectively. Intra-assay coefficient variationss were ranging from 3.0% to 3.3%, inter-assay CV-s from 3.5% to 5.4% in case of leptin ELISA. Both adiponectin and leptin are 4.5 hours ELISA with 3.9–250 ng/mL assay range and 0.891 ng/mL sensitivity (adiponectin); and 15.6–1000 pg/mL assay range and 7.8 pg/mL sensitivity (leptin), respectively.

### 2.8 Assessment of Peripheral Nerve Function

All participants were evaluated in detail for peripheral neuropathy (DN4 questionnaire in screening for neuropathic pain, vibration perception threshold, quantitative sensory testing) and *in-vivo* confocal microscopy of the cornea by ophthalmologist for the diagnosis of distal sensory polyneuropathy. Peripheral sensory nerve functions were assessed with current perception threshold testing (CPT) using the Neurometer®. It has been previously reported that Neurometer® is capable of detecting distal sensory neuropathy in various diseases, including diabetes mellitus [21]. The CPT testing using Neurometer® delivers sinusoidal alternating current stimuli at three different frequencies: 5 Hz, 250 Hz and 2000 Hz, assessing small unmyelinated C-fibre, small myelinated Aß-fibre and large myelinated Aß-fibre function, respectively. This intensity setting is performed to approach the CPT value within a ± 50 microampere range from the full range of 0 to 9.99 milliamperes [22, 23]. The current stimuli were applied unilaterally to the dorsal surface of the distal phalangus of the index finger and great toe through two small electrodes and the intensity was elevated until the subjects indicated a painless sensation. Neurometer® CPT testing is used to adjust the level of stimulation based on the patient’s response. A CPT value based on the minimal current perceived was calculated once patients gave a sufficient number of correct consecutive responses out of 5 to 7 randomly generated sets of stimuli above and below their level of perception.

### 2.9 Statistical Analyses

We performed Statistica® TIBCO Software Inc. (2018). Statistica (data analysis software system), version 13. http://tibco.com during statistical analyses. Normality of data distribution was checked with the Kolmogorov–Smirnov and Shapiro-Wilk tests. Relationship between two categorical variables is calculated by Chi-squared test. In case of normal distribution, the differences between anthropometric and clinical laboratory values in diabetic controls and patients before exercise program were analyzed with unpaired Student’s *t* test. Data were expressed as means ± standard deviation. In case of non-normal distribution, the previously mentioned differences were tested by Mann-Whitney u-test. Data are presented with median (lower-upper quartile). Differences before and after exercise program were performed with paired Student’s *t* test (normal distribution) or Wilcoxon matched paired test (non-normal distribution). Correlations between continuous parameters were investigated with Pearson’s correlation test. Values under *p *≤ 0.05 were considered statistically significant.

## 3. Results

### 3.1 Clinical and Laboratory Parameters of DN Patients before and after Physical Activity and Compared their Data to Diabetic Patients without Neuropathy

Clinical and laboratory parameters of DN patients before and after physical activity and data of controls are summarized in Table 1.

**Table 1.** **Clinical and laboratory parameters of enrolled diabetic patients**.

	(1) Diabetic patients with neuropathy before aerobic exercise	(2) Diabetic patients with neuropathy after aerobic exercise	(3) Control patients with type 2 diabetes
Number of patients (male/female)	30 (9/21)		32 (10/22)
Age of patients (years)	61.97 ± 8.09		64.37 ± 6.52
Duration of diabetes (years)	10.3 ± 3.7		10.9 ± 4.1
BMI (kg/$m^{2}$)	31.6 ± 3.94	31 ± 3.81 ^##^	29.3 ± 2.88 ^$$^
Waist circumference (cm)	92.2 ± 16.22	90.07 ± 16.04 ^##^	88.82 ± 10.68 ^$$,††^
hsCRP (mg/L)	3.95 (1.5–9.1)	3.49 (1.8–6.5) ^##^	1.4 (0.6–2.9) ^†^
HbA1C (%)	7.09 ± 0.81	6.78 ± ${\mathrm{0.87}}^{\#\,\#}$	6.98 ± 1.05
Creatinine (µmol/L)	72.73 ± 14.48	74.27 ± 18	77.44 ± 19.66
Uric acid (µmol/L)	305.57 ± 68.6	308.33 ± 67.31	300.22 ± 61.95
Alanine Aminotransferase (U/L)	34.5 ± 3.6	32.1 ± 4.2	36.7 ± 2.8
Aspartate Aminotransferase (U/L)	26.4 ± 3.7	28.9 ± 3.9	25.7 ± 3.5
Gamma-Glutamyl Transferase (U/L)	38.6 ± 4.4	36.3 ± 5.1	33.8 ± 5.2
Triglyceride (mmol/L)	2.5 ± 1.54	2.23 ± 1.09	2.3 ± 1.28
Total cholesterol (mmol/L)	4.79 ± 1.17	4.77 ± 1.18	4.8 ± 0.97
HDL-cholesterol (mmol/L)	1.27 ± 0.32	1.31 ± 0.35	1.34 ± 0.36
nonHDL-cholesterol (mmol/L)	3.65 ± 1.12	3.49 ± 1.18	3.49 ± 1.25
LDL-cholesterol (mmol/L)	3.0 ± 1.03	2.95 ± 1.01	2.76 ± 1.09
FGF21 (pg/mL)	140.62 (73.19–373.07)	168.89 (111.4–513.69) ^#^	133.54 (81.52–281.76)
TNF-alpha (pg/mL)	0.7 ± 0.4	0.57 ± 0.21 ^#^	0.59 ± 0.27 ^$^
Irisin (µg/mL)	4.32 ± 1.33	4.3 ± 1.29	4.73 ± 0.8
Adiponectin (µg/mL)	6.91 ± 3.32	7.09 ± 3.88	6.89 ± 3.32
Leptin (ng/mL)	30.72 ± 19.98	30.59 ± 18.38	20.93 ± 18.97 ^$, †^
Current perception threshold (by Neurometer®, mA)	0.545 ± 0.082	0.498 ± 0.088 ^#^	0.458 ± 0.021 ^$$,†^

Data are presented as mean ± SD or median (interquartile ranges). Abbreviations: BMI, body mass index; FGF21, fibroblast growth factor-21; HbA1c, haemoglobin A1c; HDL, high-density lipoprotein; hsCRP, high sensitivity C-reactive protein; LDL, low-density lipoprotein; TNF-alpha, tumor necrosis factor-alpha. #: *p *< 0.05 in DN patients before and after physical activity. ##: *p *< 0.005 in DN patients before and after physical activity. $: *p *< 0.05 in DN patients before physical activity compared to diabetic controls. $$: *p *< 0.005 in DN patients before physical activity compared to diabetic controls. †: *p *< 0.05 in DN patients after physical activity compared to diabetic controls. ††: *p *< 0.005 in DN patients after physical activity compared to diabetic controls.

Significant decreases in BMI, waist circumference, hsCRP, HbA1c and TNF-alpha levels were observed after 6-week physical activity in DN patients. Circulating FGF21 levels were significantly increased; while CPT values as measured by Neurometer® test were significantly improved after 6-week physical activity in DN patients.

There were no differences in serum creatinine, uric acid, irisin, adiponectin, leptin, triglyceride, total cholesterol, HDL-C, nonHDL-C, LDL-C levels and enzyme liver parameters in DN patients before and after physical activity.

Circulating TNF-alpha and hsCRP was significantly higher in DN patients compared to controls (Table 1). Although the concentrations of adipocytokines did not change across groups after physical activity, the level of leptin was significantly decreased after physical activity compared to control subject.

### 3.2 Correlations between Clinical and Biochemical Parameters and Change of FGF21 during Physical Activity

**Fig. 2.**
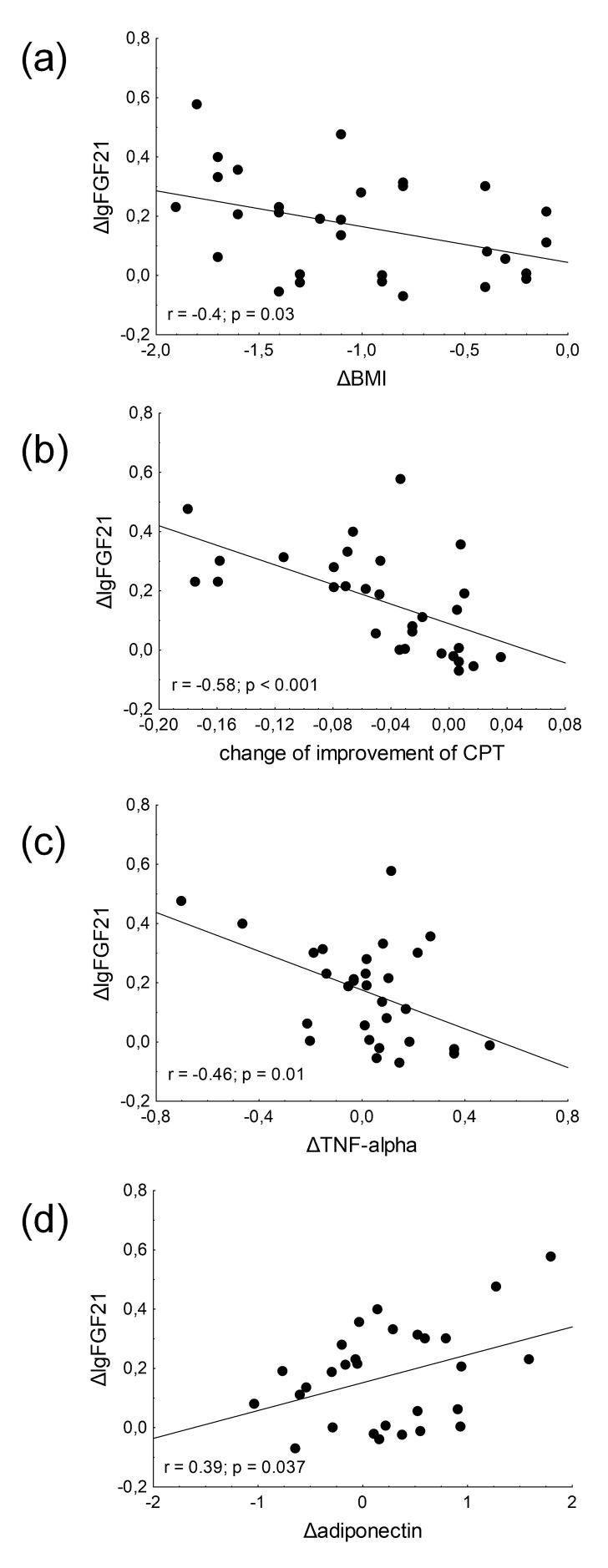
**Correlations between changes of fibroblast growth factor 21 (FGF21) levels and (a) change of body mass index (BMI); (b) change of improvement of CPT; (c) changes of tumor necrosis factor alpha (TNF-alpha) levels and (d) changes of adiponectin levels in type 2 diabetic patients with neuropathy**. Pearson’s correlation test was performed to investigate the relationship between continuous variables.

Significant negative correlations were observed between the changes in FGF21 levels and BMI (r = –0.4, *p* = 0.03) (Fig. 2a), between changes in FGF21 and the improvement of CPT values (r = –0.58, *p *< 0.001) (Fig. 2b) and between the changes in FGF21 and TNF-alpha levels (r = –0.46, *p* = 0.01) in DN patients after 6-week physical activity (Fig. 2c). We found significant positive correlation between the changes in the levels of adiponectin and FGF21 (r = 0.39, *p* = 0.037) in DN patients after physical activity (Fig. 2d).

We found a significant positive correlation between changes in BMI and TNF-alpha concentrations (r = 0.39, *p *< 0.05) in DN patients after physical activity (data not shown).

Significant negative correlation was observed between the changes in BMI and adiponectin levels (r = –0.38, *p *< 0.05). There was no association between changes in the levels of TNF-alpha or FGF21 and changes in HbA1c levels in DN patients (data not shown).

### 3.3 Correlations between Changes of Clinical Parameters and Cytokines, Adipokines and Myokines During Physical Activity

We found a significant positive correlation between changes in CPT values and TNF-alpha concentrations (r = 0.62, *p *< 0.001) and an inverse correlation between changes in CPT values and adiponectin levels (r = –0.4, *p *< 0.05) (data not shown). We did not detected any correlation between changes in CPT values and irisin levels.

## 4. Discussion

To our knowledge, this is the first study, to demonstrate significant correlations between the change of FGF21 concentration and change of body mass index, as well as change of TNF-alpha and adiponectin levels in T2DM patients with peripheral neuropathy after six weeks of aerobic exercise. In addition, we firstly analyzed the change in FGF21 levels in diabetic neuropathy and found a significant increase after training program.

In our study, we found a significant increase in FGF21 levels in type 2 diabetic patients after 6 weeks of physical activity. Previous research has also shown that physical activity may increase the serum level of FGF21 in T2DM but neuropathy as a microvascular complication has not been reported in these studies [7, 24]. A recent retrospecive study also revealed that the levels of serum FGF21 were elevated in patients with higher BMI compared to individuals with normal or low BMI. Moreover, the FGF21 concentrations were found to be higher in patients who exercised regularly compared to those who exercised only intermittently or not at all [25]. our result may be explained by the activating effect of FGF21 on FGFR1 receptor and β-Klotho cofactor inducing the oxidation of fatty acids and the inhibition of lipogenesis. The circulating FGF21 acts through cell surface receptors comprised of FGF receptors (FGFR) in complex with transmembrane protein β-Klotho. The interaction of FGFR with β-Klotho increases sensitivity to FGF21 and the ability to activate intracellular signaling pathways, which eventually leads to metabolic effects [9]. Experimental studies have also shown that treatment with FGF21 induces weight loss, increases insulin sensitivity and lowers triglyceride levels in obese animal models. Overall, these findings supported a beneficial effect on body weight and fat distribution, and both white and brown adipocytes express high levels of β-Klotho and FGFR1c, a member of the FGFR family, consistent with sensitivity to FGF21 in adipose tissue [6, 26]. Plasma level of FGF21 is induced lipolysis in adipose tissue, especially via activation of hormone sensitive lipase and adipose triglyceride lipase [27]. Futher research has shown that FGF21 knockout mice exhibited decreased fasting blood glucose level, glucogenesis, liver beta-oxidation and ketogenesis demonstrating that FGF21 mediated the effect of peroxisome proliferator-activated receptor-alpha during the adaptation to fasting and exercise in skeletal muscle [28]. In insulin resistance, mitochondrial respiratory chain deficiency associated with a compensatory response in skeletal muscle cells via increased expression and decreased degradation of FGF21 mRNA results in enhanced mitochondrial function through a PGC-1a dependent pathway [29]. PGC-1α is a major regulator of mitochondrial biogenesis, by upregulating nuclear respiratory factor and mitochondrial transcription factor A, leading to an overall increase in mitochondrial DNA replication and gene transcription. FGF21 knockout mice fail to induce PGC-1a expression in response to a prologned fast and have impaired gluconeogenesis and ketogenesis [28]. We hypothesized that an increase in FGF21 level may induce lipolysis in adipose tissue, especially via activation of hormone sensitive lipase and adipose triglyceride lipase, and enhance mitochondrial function as a result of physical training T2DM subjects with neuropathy. Further prospective studies will be necessary to confirm these results and explore mechanisms in future research.

Growing evidence suggests that FGF21 may reduce atherosclerosis in cardiovascular disease [30, 31]. Recent research has demonstrated that FGF21 may inhibit arterial calcification in experimental models of vascular injury via various mechanisms including suppression of endoplasmic reticulum stress-mediated apoptosis and inhibiting the osteogenic transition of vascular smooth muscle cells [32]. Paradoxically, there is a positive association between FGF21 levels and a number of cardiovascular or metabolic diseases, such as coronary heart disease, obesity and T2DM [33, 34]. On the other hand, based on another clinical trial, acute myocardial infarction was also associated with a decrease in circulating FGF21 levels [35]. Thus, a number of studies on this topic have been published with contradictory results in recent years, which have often shown inconsistencies and contradictions between studies in terms of metabolic parameters and medication of patients. The results of our study in patients with DN are in line with previous studies showing that exercise may increase the serum levels of FGF21 not only in patients with T2DM, but also in distal sensory polyneuropathy [7, 24].

It must be noted that there are no data on the effects of aerobic exercise on FGF21 levels in subjects with distal sensory polyneuropathy. This is the first report on beneficial effect of physical activity on FGF21 levels in distal sensory polyneuropathy that strengthens the beneficial effects of physical exercise on sensory symptoms and neuropathic deficits in T2DM patients. The change of FGF21 level correlated with the severity of peripheral sensory neuropathy—defined by Neurometer®—after physical activity. Therefore, increased serum FGF21 levels may predict the clinical response to aerobic exercise. Previous studies have demonstrated a significant improvement in neurological function, affecting both sensorimotor and autonomic components of the peripheral nervous system, in patients with DN during physical exercise programmes [2, 36]. The mechanism of improvement of neuropathic symptoms and nerve function is thought to be related to improvement in endothelial dysfunction and reduction of inflammation in DN. Our data highlight the initial hypothesis that increased FGF21 may be a biomarker of chronic inflammation in DN. However, there are no previous data in the literature on whether physical activity directly or indirectly elevates FGF21 in patients with diabetic neuropathy. Therefore, further studies are necessary to validate our results.

Although the levels of adipokines did not differ in patients with diabetic neuropathy after physical activity, the increasing tendency in adiponectin level was significantly associated with the magnitude of body weight loss and we found a positive correlation between the increase in the adiponectin level and the FGF21 concentration in diabetic neuropathy. FGF21 shows functional similarity to adiponectin, which acts as a downstream effector of FGF21, controling glucose and lipid metabolism in adipocytes and skeletal muscle [37]. Meanwhile, adiponectin may enhance the effect of FGF21 on energy balance and insulin sensitivity in these tissue; thus, the FGF21— adiponectin axis may be implicated in the regulation of glucose and lipid homeostasis [38]. We have also found a significant correlation between the level of serum adiponectin and improvement of neurological function, affecting sensorimotor component of the peripheral nervous system in patients with diabetic neuropathy. Previous research has been reported that decreased adiponectin levels were associated with a significantly increased risk of DN in T2DM patients. Moreover, there was a strong relationship between decreased nerve conduction velocity and adiponectin concentration in chronic inflammation and progression of diabetic sensorimotor neuropathy [39].

Our study revealed that six weeks of aerobic physical activity in patients with DN lead to a significant reduction in the levels of TNF-alpha and hsCRP. Recent studies have demonstrated the efficacy of physical exercise on inflammatory markers in DN [36, 40]. TNF-alpha plays a crucial role in initiating inflammatory processes leading to severe impairment of glucose tolerance and insulin sensitivity which may eventually increase the risk of cardiovascular diseases in T2DM [41]. TNF-alpha stimulates lipolysis in adipose tissue, thus increased plasma concentration of FFA may contribute to atherogenesis in T2DM patients. Moreover, TNF-alpha enhances leptin production, which is known to regulate energy homeostasis by reducing pancreatic insulin secretion and promoting insulin resistance. Therefore, TNF-alpha may indirectly contribute to the development of insulin resistance by inhibiting adiponectin and stimulating leptin via glucose metabolic pathways [41]. Our findings regarding the linear association between the changes of body mass index and TNF-alpha levels were generally consistent with prior research in patients with DN after exercise program [36, 40]. However, aerobic training may have a protective effect against diabetic nerve damage by restoring endothelial function and decreasing the production of the inflammatory cytokine TNF-alpha in T2DM.

While some prior studies have demonstrated positive association or contradictory results, we have not found association between the changes in irisin levels after physical activity. Previous research has shown that FNDC5 expression in skeletal muscle are reduced in obese subjects and circulating irisin levels are related with insulin sensitivity in T2DM [11]. Studies examining the relationship between the circulating irisin levels and training-induced changes have yielded mixed results, with some studies suggesting a strong association and others finding no association [42, 43, 44]. It was hypothesized that the level of serum irisin increased immediately after physical activity and seems to correlate with the intensity of exercise training, as well as prior empirical research suggest the contribution of irisin in the neuroprotective process of physical exercise in T2DM [45, 46]. Therefore, further follow-up studies should be performed to determine the effects of various factors directly or indirectly for changes in levels of irisin in T2DM with peripheral neuropathy.

There are some limitations of our study. Data on other inflammatory biomarkers and parameters of endothelial dysfunction, arterial stiffness and flow mediated dilatation would enhance our knowledge about the impactt of physical activity on chronic inflammation and endothelial dysfunction and its contribution to the favorable effects on peripheral neuropathy. Evaluation of sural nerve automated nerve conduction in the diagnosis of peripheral neuropathy could be useful additional information. Direct measurement on ${\mathrm{VO}}_{\text{2max}}$ has been used as the gold standard test of cardiovascular fitness. In our study, an indirect field test was used in which case the value of VO2 may be slightly overestimated in study population. The Neurometer©, a type of current output sensory nerve conduction threshold test, is available for the diagnosis and follow-up of peripheral sensory nerve conditions in diabetes mellitus. However, electroneurography is the current gold standard test for diagnosis of polyneuropathy. We concluded that monitoring of FGF21 levels may predict the efficacy of aerobic exercise in diabetic neuropathy, but other lifestyle factors such as changes in eating habits or lifestyle-oriented motivations might also have contributed.

## 5. Conclusions

Our results highlight the potential role of regular physical activity in the treatment of diabetic neuropathy. According to our results, physical activity increased the levels of FGF21 in T2DM patients with distal sensory polyneuropathy. Monitoring of FGF21 levels may predict the efficacy of aerobic exercise in diabetic neuropathy. Data on other biomarkers of inflammation and oxidative stress may enhance our knowledge about the impact of physical activity in peripheral sensorimotor neuropathy.
